# Examining the Effects of Environment, Geography, and Elevation on Patterns of DNA Methylation Across Populations of Two Widespread Bumble Bee Species

**DOI:** 10.1093/gbe/evae207

**Published:** 2024-09-27

**Authors:** Sam D Heraghty, Sarthok Rasique Rahman, Kelton M Verble, Jeffrey D Lozier

**Affiliations:** Department of Biological Sciences, The University of Alabama, Tuscaloosa, AL, USA; Department of Biological Sciences, The University of Alabama, Tuscaloosa, AL, USA; Department of Ecology and Evolutionary Biology, 106A Guyot Hall, Princeton University, Princeton, NJ 08544; Department of Biological Sciences, The University of Alabama, Tuscaloosa, AL, USA; Department of Biological Sciences, The University of Alabama, Tuscaloosa, AL, USA

**Keywords:** Bombus, methylation, landscape genomics, environmental association, epigenetics

## Abstract

Understanding the myriad avenues through which spatial and environmental factors shape evolution is a major focus in biological research. From a molecular perspective, much work has been focused on genomic sequence variation; however, recently there has been increased interest in how epigenetic variation may be shaped by different variables across the landscape. DNA methylation has been of particular interest given that it is dynamic and can alter gene expression, potentially offering a path for a rapid response to environmental change. We utilized whole genome enzymatic methyl sequencing to evaluate the distribution of CpG methylation across the genome and to analyze patterns of spatial and environmental association in the methylomes of two broadly distributed montane bumble bees (*Bombus vancouverensis* Cresson and *Bombus vosnesenskii* Radoszkowski) across elevational gradients in the western US. Methylation patterns in both species are similar at the genomic scale with ∼1% of CpGs being methylated and most methylation being found in exons. At the landscape scale, neither species exhibited strong spatial or population structuring in patterns of methylation, although some weak relationships between methylation and distance or environmental variables were detected. Differential methylation analysis suggests a stronger environment association in *B. vancouverensis* given the larger number of differentially methylated CpG's compared to *B. vosnesenskii*. We also observed only a handful of genes with both differentially methylated CpGs and previously detected environmentally associated outlier SNPs. Overall results reveal a weak but present pattern in variation in methylation over the landscape in both species.

**Significance**

Variation in patterns of methylation across the landscape has not yet been extensively studied in bumble bees. Here, we provide the first study to do so in 2 different species of bumble bee, *Bombus vancouverensis* Cresson and *Bombus vosnesenskii* Radoszkowski. These species have different environmental niche breadths with *B. vosnesenskii* having a wider niche than *B*. *vancouverensis*, especially for temperature. We find that there are landscape level patterns in methylation variation in both species, although these patterns are fairly weak. Between the two species, the environment has a stronger influence on methylation variation in *B. vancouverensis* than in *B. vosnesenskii*, which mirrors previous whole genome work and adds more evidence that niche breadth can affect the path of genome evolution within species.

## Introduction

The increasing availability of genomic data has led to an explosion of research examining the effect of spatial and environmental factors on molecular evolution in natural landscapes (Storfer et al. 2018; Dorant et al. 2022). To date, most studies have focused on how DNA sequence-based variation such as single nucleotide polymorphisms (SNPs) or structural variants such as small indels are associated with environmental variables (Ahrens et al. 2018; Cayuela et al. 2021; Hartke et al. 2021). However, increasing attention is being given to the role of epigenetic variation as a factor that can shape species responses to environmental pressures (Mccaw et al. 2020; Gao et al. 2022; Carvalho 2023). Epigenetics refers to a suite of mechanisms that can change gene expression without altering the sequence of DNA itself and includes noncoding RNAs, histone modification, and DNA methylation. DNA methylation, or the addition of a methyl group (CH_3_) to a cytosine in certain contexts, has received much attention because DNA methylation has been shown to alter gene expression and because methylation patterns can change during an organism's lifespan (Harrison et al. 2022; Nakamura et al. 2023). There is also some evidence that methylation patterns can shift in response to environmental conditions, which may ultimately represent a possible mechanism for tolerating rapid climate changes (Chano et al. 2021; Gupta and Nair 2022; Carvalho 2023). For example, there is evidence in corals that changes in methylation may be adapting to variable thermal conditions (Dixon et al. 2018; Rodríguez-Casariego et al. 2020). Additionally, methylation may act to guide mutation of genes that yield adaptative phenotypes in response to certain environmental stimuli (Flores et al. 2013). Work in *Daphnia* exposed to pollution also identified changes in DNA methylation that may help populations persist (Harney et al. 2022), which suggests that DNA methylation maybe useful across a variety of different environmental stressors.

DNA methylation is a common form of epigenetic modification; however, methylation may serve different roles in different lineages. For instance, mammalian genomes tend to be highly methylated (70% to 80% of CpG's), with consistently high methylation save for near promoters (Sharif et al. 2010; Li and Zhang 2014) and methylation has a role in gene silencing (Smith and Meissner 2013). Alternatively in arthropods, methylation is much less frequent [∼0% to 14% of CpG's, with most methylation in gene bodies (Bewick et al. 2017; Lewis et al. 2020)], and the purpose of methylation is less clear, although unlike mammals highly methylated genes can be expressed (Lewis et al. 2020). There is mixed evidence for DNA methylation being involved in both alternative splicing [noted in mealybugs, ants, and honeybees (Bonasio et al. 2012; Li-Byarlay et al. 2013; Bain et al. 2021)], immune response in honey bees (Li-Byarlay et al. 2020), and the evolution of sociality (Yan et al. 2015; Bewick et al. 2017). Thus, more work is needed to better understand DNA methylation in arthropods, including how patterns vary within and among species.

Species with broad geographic ranges that encompass substantial environmental heterogeneity can be tools to understand mechanisms of adaptation or plasticity that can be shaped by local environmental pressures. Species with montane distributions may be especially useful as differences in environmental conditions can emerge at both small and large spatial scales (Rahbek et al. 2019a, 2019b). Bumble bees (Hymenoptera: Apidae: *Bombus*) are a globally distributed genus of insects that are ecological and economically important pollinators (Greenleaf and Kremen 2006; Strange 2015; Cameron and Sadd 2020). There has been much research on potential environmental adaptation in bumble bees using a variety of different genomic (Kent et al. 2018; Theodorou et al. 2018; Sun et al. 2020; Heraghty et al. 2022) and transcriptomic approaches (Pimsler et al. 2020; Liu et al. 2020a; Liang et al. 2022). However, there has been limited work studying how DNA methylation is involved in environmental adaptation (Dillon and Lozier 2019; Rahman and Lozier 2023). Most studies of methylation in bumble bees have been involved in understanding possible roles of methylation in sociality and caste development (Lockett et al. 2016; Li et al. 2018; Bain et al. 2021) although there are some exceptions, like work examining the effect of neonicotinoids on methylation (Bebane et al. 2019). Despite the relatively low levels of methylation in bumble bees (∼1% of CpG's) (Marshall et al. 2019, 2023; Pozo et al. 2021; Rahman and Lozier 2023), variable CpG methylation still may have a role in coping with environmental stress. Prior work in *Bombus* found that colony identity better explains individual methylation patterns than social caste, which suggests genomic background strongly influences methylation patterns (Marshall et al. 2019). Therefore, it is possible that selection could act on the genome to change methylation patterns, which in turn could affect gene expression or other processes.

**Fig. 1.**
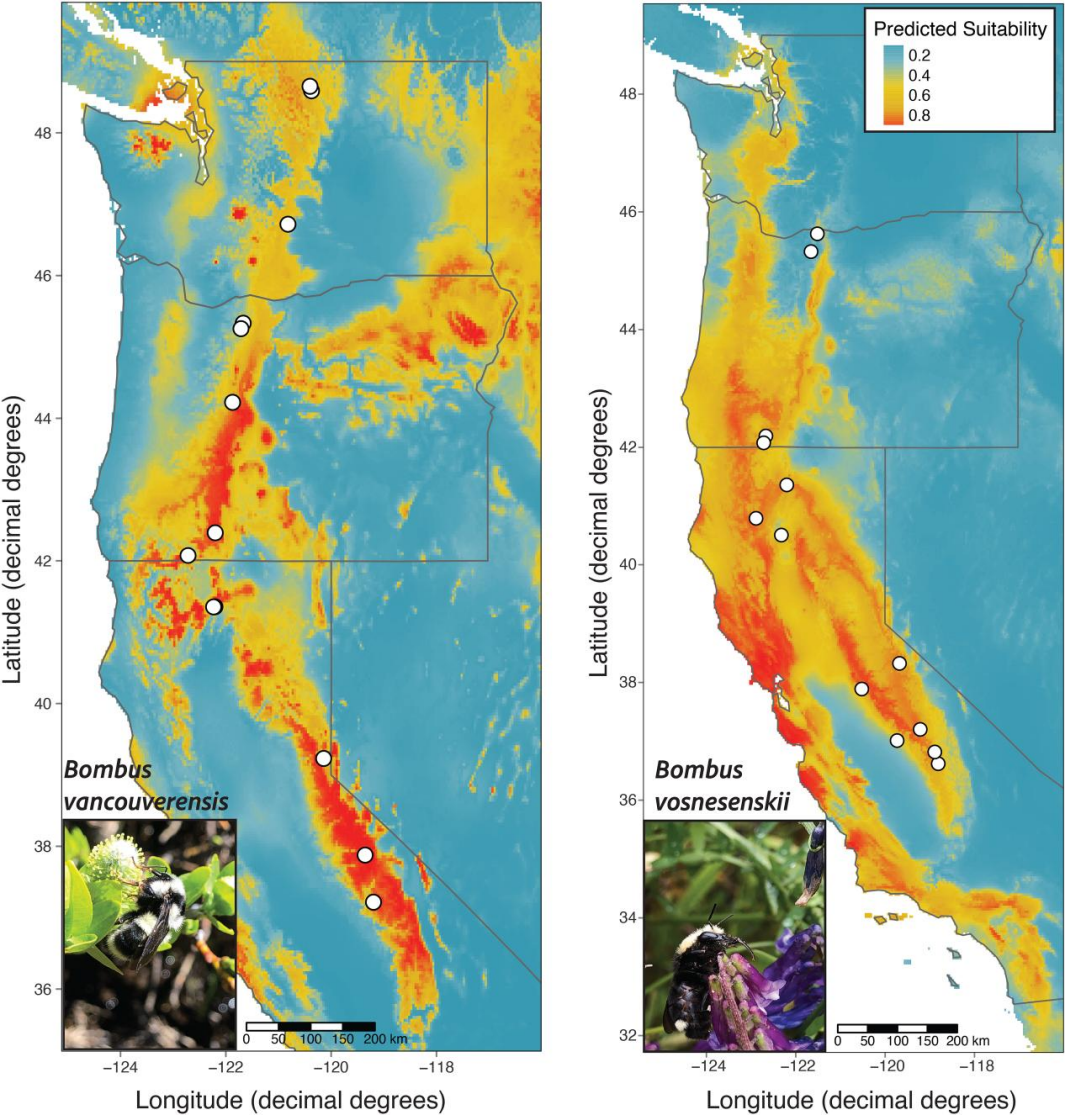
Maxent range map of both species (*Bombus vancouverensis* [left] and *Bombus vosnesenskii* [right]; photos on inset) using presence data from Cameron et al. (2011). Circles indicate sampling localities. The *y* axis indicates degrees latitude and the *x* axis indicates degrees longitude.

*Bombus vancouverensis* Cresson and *Bombus vosnesenskii* Radoszkoswki are two species of common bumble bee found in western North America (Fig. 1, Cameron et al. 2011). These two species have partially overlapping ranges in California, Oregon, and Washington, United States, but also have some unique aspects to their distributions. *Bombus vancouverensis* has a broader geographic range across the western United States and Canada, but in the west coast states is generally associated with narrower distributions of several environmental variables (especially temperature), whereas *B. vosnesenskii* has a smaller geographic range but appears to have a more flexible niche that allows persistence across a wider range of habitats and environmental conditions in the region (Jackson et al. 2018). For example, although *B. vancouverensis* is observed across a broad range of elevations, because of a preference for cooler temperatures its elevational breadth is dependent on latitude, where bees in the southern parts of the range (e.g. Sierra Mountains in southern California) are restricted to high elevations and northern populations can be found closer to sea level. In contrast, *B. vosnesenskii* can be found at a broader range of elevations throughout its distribution (Koch et al. 2012; Jackson et al. 2018). Several studies have examined these two species to gain insight into similarities and differences in their ecology and evolution, and in most cases *B. vancouverensis* tends to show clearer and more consistent associations in traits and genetic variation with spatial–environmental gradients. For example, *B. vancouverensis* exhibits greater population structure compared to near-panmixia in *B. vosnesenskii* (Jackson et al. 2018; Heraghty et al. 2023, 2022), *B. vancouverensis* exhibits greater variation in morphological traits associated with elevation (Lozier et al. 2021), and whole genome studies have found stronger signals of environmental association across the genome of *B. vancouverensis* (Heraghty et al. 2023, 2022). Evaluating differences in methylation across the range of both species will be a useful addition to further understand how differences in species distributions and demography can influence molecular evolution.

In this study, we aim to build on prior genomic work on DNA sequence variation across the *B. vancouverensis* and *B. vosnesenskii* ranges by characterizing genome-wide 5′-CpG-3′ DNA methylation from broad latitudinal and altitudinal gradients. We then focus on comparing the overall distribution of genome-wide CpG methylation of each species to test for spatially or environmentally associated methylation that might reveal consistent differences in methylation among populations. Overall, we seek to identify and compare landscape level patterns in DNA methylation between a species with weak gene flow that occupies a broader range of environmental conditions (*B. vosnesenskii*) and a species with stronger population structure and tends to be distributed in narrower climatic zones at relatively high elevations in the region (*B. vancouverensis*). Specifically, we aim to test the hypothesis that DNA methylation variation will parallel previous genomic results by exhibiting stronger spatially and environmentally associated differentiation in *B. vancouverensis*.

## Results

### Sequencing Data Summary

A total of 53 and 54 female workers from 13 unique localities each for *B. vancouverensis* and *B. vosnesenskii* were used for whole-genome enzymatic methylation sequencing (Fig. 1, supplementary table S1, Supplementary Material online). Sequencing produced 27,750,092.6 (±5,219,102.81 SD) paired reads per sample for *B. vancouverensis* (∼14× coverage) and 43,544,649.6 (±14,655,370.3 SD) reads per sample for *B. vosnesenskii* (∼22× coverage). After filtering for *B. vancouverensis*, 11,351,115 total CpG's (both methylated and unmethylated) were retained for the full CpG set, with 11,161,598 retained (Table 1) after removal of SNPs from previous range-wide whole genome resequencing (Heraghty et al. 2022). For *B. vosnesenskii*, 26,120,126 CpG sites were retained in the full set, with 23,170,123 (Table 1) retained after SNP removal (Heraghty et al. 2023). This difference between species was driven by sequencing depth differences leading to fewer CpGs passing filters in *B. vancouverensis*. The average percent methylation was 0.783% and 0.777% across all CpG sites in *B. vancouverensis* and *B. vosnesenskii*, respectively, matching results indicating low CpG methylation levels in other bumble bees (Marshall et al. 2019, 2023; Pozo et al. 2021; Rahman and Lozier 2023). A total of 327,915 and 601,896 SNPs were called by BISCUIT (Zhou 2024) from the enzymatic methyl-seq data for *B. vancouverensis* and *B. vosnesenskii* respectively.

**Table 1:** The number of CpG's found in each dataset for each species after the appropriate filters were applied

	*B. vancouverensis*	*B. vosnesenskii*	Criteria
Total CpGs	11,351,115	26,120,126	All CpGs (methylated and unmethylated) retained after basic filtering
Total CpGs with SNP filter	11,161,598	23,170,123	All CpGs (methylated and unmethylated) retained after basic filtering + SNP filter
CpGs in Meth30	87,673	194,247	CpG's with a minimum average methylation value of 30% across all individuals
CpGs in Meth30 with SNP filter	82,834	172,333	CpG's with a minimum average methylation value of 30% across all individuals + SNP filter
CpGs in Hvar	270,025	669,523	CpGs with methyation > 2 SD + have nonzero methylation in at least 4 individuals
CpGs in Hvar with SNP filter	255,043	582,942	CpGs with methyation > 2 SD + have nonzero methylation in at least 4 individuals + SNP filter

The criteria column details the filtering criteria used for each dataset (see Materials and Methods for details)

### Distribution of Methylated CpGs within the Genome

**Fig. 2.**
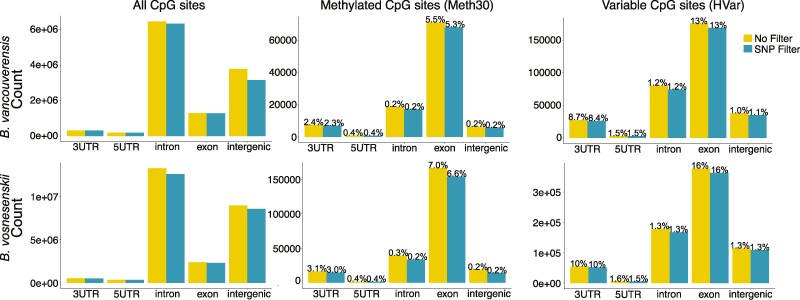
The distribution of CpG's in genomic features across all sequenced CpG's, methylated CpG's, and variables CpG's both with and without SNP filtering. The height of the bar indicates the total number of CpG's found in a given feature for a given dataset. Numbers indicate the portion of sequenced CpG's located in that feature that was methylated.

Of the total CpGs, 87,673 (0.79%) and 194,247 (0.84%) were retained (Table 1) in the dataset containing CpG's with at least 30% methylation averaged across all individuals (Meth30 dataset) for *B. vancouverensis* and *B. vosnesenskii*, respectively. In the high variability HVar dataset (CpG's with methylation values with >2 SD and that are called as methylated in at least 4 individuals), 270,025 and 669,523 CpGs were retained (Table 1) for *B. vancouverensis* and *B. vosnesenskii*, respectively. The SNP filter excluded a relatively small number of methylated CpGs in *B. vancouverensis* [4,839 (5.2%) and 14,982 (5.2%) from Meth30 and HVar datasets, respectively (Table 1)], with a slightly higher proportion of CpGs excluded in *B. vosnesenskii* [21,914 (10.1%) and 86,581 (12.9%) sites from Meth30 and HVar datasets (Table 1)]. The majority of CpG sites sequenced (methylated and unmethylated) were in introns and intergenic regions (Fig. 2). However, the distribution of methylated CpGs was heavily biased to genic regions in both species (e.g. 87% in *B. vancouverensis* and 83% for *B. vosnesenskii* in the SNP filtered variable dataset), especially exons (Fig. 2, supplementary table S2, Supplementary Material online). The differences in distribution are significant based on Chi-square analysis which found significant differences (*P* < 2.2e−16) in all comparisons (supplementary table S2, Supplementary Material online).

### Methylome-wide Variation Across the Landscape

**Fig. 3.**
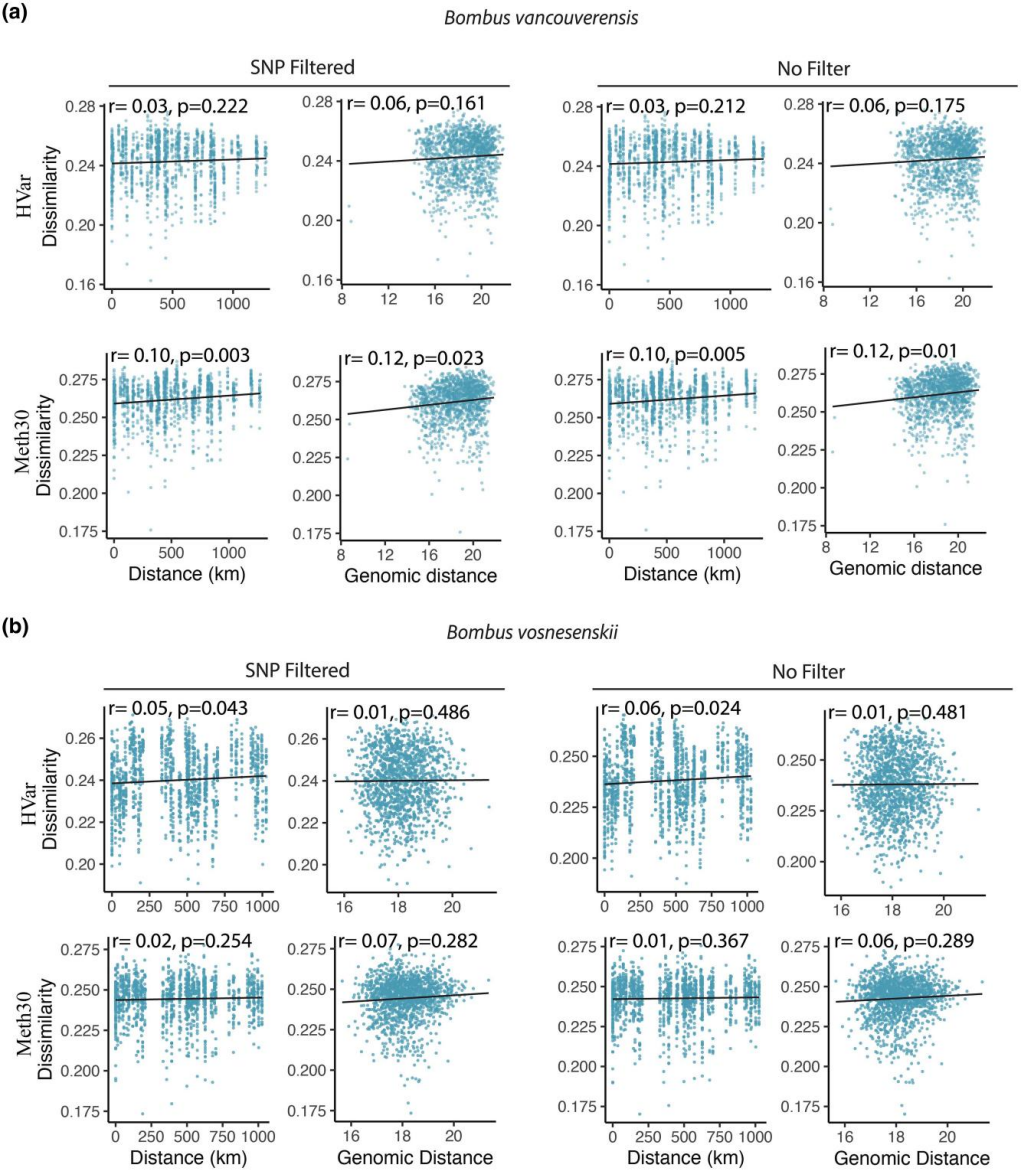
Relationship between methylation dissimilarity and both geographic and genomic distance across the HVar and Meth30 datasets as well as with and without SNP filtering for *B. vancouverensis* (a) and *B. vosnesenskii* (b). Results of the Mantel test for each relationship are printed with the corresponding scatter plot.

Using the SNPs generated by BISCUIT we detected substantial isolation by distance in *B. vancouverensis* (Mantel *r* = 0.57, *P* = 0.001) but not *B. vosnesenskii*, indicating the sequencing data for the individuals used in this study recovers the previously reported results of relatively strong population structure in *B. vancouverensis* and weak structure in *B. vosnesenskii* (Jackson et al. 2018; Heraghty et al. 2022) (supplementary fig. S1, Supplementary Material online). Both species exhibited a positive relationship between methylation dissimilarity and both geographic and genetic distance, and although some correlations were significant, overall relationships were weak and depended on filtering criteria (e.g. HVar vs. Meth30 and SNP filter vs. no filter) (Fig. 3). The strongest relationships were observed in *B. vancouverensis* when comparing methylation dissimilarity against geographic (Mantel *r* = 0.10, *P* = 0.003) and genomic distance (Mantel *r* = 0.12, *P* = 0.01) for the Meth30 dataset that focused on more highly and consistently methylated CpG's, although similarly positive but insignificant relationships were also observed in the HVar dataset (Fig. 3). For *B. vosnesenskii*, a significant, albeit weak, relationship was detected only for the SNP-filtered HVar dataset and geographic distance (Fig. 3a). As for SNP-based population structure, the overall trends in methylation suggest that *B. vancouverensis* has stronger “methylation population structure” than *B. vosnesenskii*, although even in *B. vancouverensis,* methylation dissimilarity does not approach the degree of population-level SNP differentiation observed for this species (supplementary fig. S1, Supplementary Material online; Heraghty et al. 2022).

**Fig. 4.**
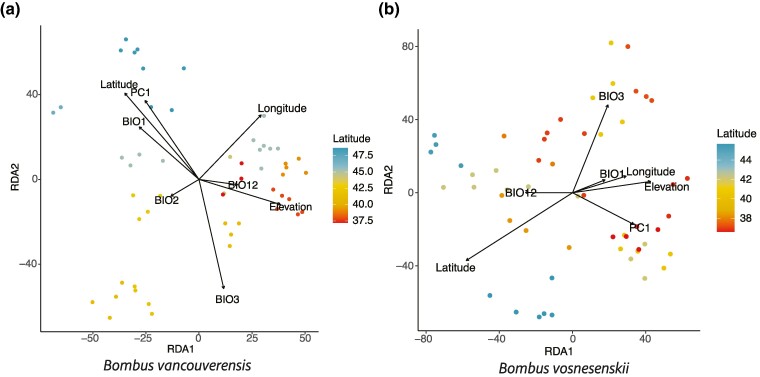
Results of full RDA model for a) *B. vancouverensis* and b) *B. vosnesenskii* using the SNP filtered dataset for both species with individuals colored by the latitude of their sampling locality. The following abbreviations are used for the BioClim variables: BIO1, annual mean temperature; BIO2, mean diurnal range; BIO3, isothermality; BIO12, annual precipitation; PC1, PC1 axis from a PCA analysis of SNP data to represent population structure.

We used redundance analysis (RDA) to identify the variation in overall methylation patterns in each sample that could be explained by environmental, spatial, and population structure variables (PC1). The difference between species was somewhat clearer when visualized using the RDAs. For *B. vancouverensis*, the full RDA model [model containing all predictors: BioClim climate variables, elevation, latitude, longitude, and PC1 from a principal component analysis (PCA) of BISCUIT SNPs to account for population structure] showed samples clustering broadly into northern and southern groups largely along RDA2 and some effects for specific variables. The PC1 and mean annual temperature (BIO1) were largely similar to latitude, which was expected given that temperature is generally inversely related to latitude and the previously identify population structure being on a latitudinal gradient (Jackson et al. 2018; Heraghty et al. 2022). Samples also loosely clustered along the elevation vector, most clearly apparent for the positively loading southern higher elevation samples along RDA1 (Fig. 4), similar to SNP results (Heraghty et al. 2022). The full RDA model was also significant and showed some latitudinal separation (largely along RDA1) for *B. vosnesenskii*. Most of the other variables also were loading along RDA1, although isothermality (BIO3) was primarily loading along RDA2. For both species, the full models were able to account for similar amounts of observed variation with 20.7% and 17.2% of variation explained for *B. vancouverensis* and *B. vosnesenskii*, respectively. Of the partial models, the environmental model (BioClim variables and elevation) performed best for both species, accounting for 62.1% and 58.0% of the explainable variance for *B. vancouverensis* and *B. vosnesenskii*, respectively (Table 2). The partial model accounting for geography (latitude and longitude), while significant, accounted for much less of the explainable variation (∼5% for both species) and the population structure (PC1 from PCA of SNP data) model was insignificant in both species. There was minimal confounded variation (∼1% of explainable variation). SNP filtering had a negligible impact (supplementary table S3, Supplementary Material online).

**Table 2:** Summarized results of pRDA data for both focal species

Model	Inertia	*R* ^2^	*P*(>F)	Proportion of explainable variance	Proportion of total variance
*B. vancouverensis*					
Full model	10,591,543	0.0205	**0**.**001**	1	0.207
Environment	6,575,573	0.0129	**0**.**001**	0.621	0.128
Geography	2,728,061	0.00727	**0**.**001**	0.258	0.053
Structure	1,188,863	0.000443	0.424	0.112	0.023
Confounded	99,046	…	…	0.009	0.002
Total unexplained	40,675,527	…	…	…	0.793
Total inertia	51,267,070	…	…	…	1
*B. vosnesenskii*					
Full model	13,356,586	0.0167	**0**.**001**	1	0.172
Environment	7,739,843	0.0114	**0**.**001**	0.579	0.01
Geography	3,810,203	0.00521	**0**.**005**	0.285	0.049
Structure	1,664,682	−0.000315	0.614	0.125	0.021
Confounded	141,858	…	…	0.011	0.002
Total unexplained	64,388,098	…	…	…	0.828
Total inertia	77,744,684	…	…	…	1

Inertia is synonymous with variance. Model significance is reported in the *P*(>F) column with significant models (*P* < 0.05) being denoted by bold text. Proportion of explainable variance is the ratio between the inertia of a given model and the full model. The proportion of total variance is the ratio between inertia accounted for in a given model and the total inertia in the dataset.

### CpG-level Differential Methylation

Analysis of CpGs that were differentially methylated in association with specific environmental variables identified more significantly differentially methylated CpGs (*q* ≤ 0.05) in *B. vancouverensis* than in *B. vosnesenskii* (Table 3). In *B. vancouverensis*, the largest number of differentially methylated CpG's were found to have a statistically significant associated with elevation (280 and 300 for SNP filtered and unfiltered respectively, Table 3). Both latitude (*n* = 108 and *n* = 124 for SNP filtered and unfiltered, respectively) and annual mean temperature (BIO1) (*n* = 92 and *n* = 97 for SNP filtered and unfiltered, respectively) also were associated with relatively large numbers of differentially methylated CpG's. For *B. vosnesenskii*, the largest number of differentially methylated CpG's was associated with latitude in the unfiltered dataset (*n* = 30). In the SNP-filtered dataset, the largest number of differentially methylated CpG's was associated with BIO3 (*n* = 15). Few CpG's were associated with BIO1 (*n* = 0 and *n* = 4 for SNP-filtered and unfiltered, respectively) or annual precipitation (BIO12) (*n* = 3 and *n* = 6 for SNP-filtered and unfiltered, respectively).

**Table 3:** Summary of the number of individual CpGs identified as differentially methylated with each variable using either the filtered or not filtered dataset (FDR corrected *q* < 0.05)

	No filter		Filter	
Variable	*B. vancouverensis*	*B. vosnesenskii*	*B. vancouverensis*	*B. vosnesenskii*
Annual mean temp. (BIO1)	97	4	92	0
Mean diurnal range (BIO2)	20	N/A	21	N/A
Isothermality (BIO3)	33	12	21	15
Annual precipitation (BIO12)	42	6	52	3
Elevation (m)	300	14	280	10
Latitude	124	30	108	9

**Fig. 5.**
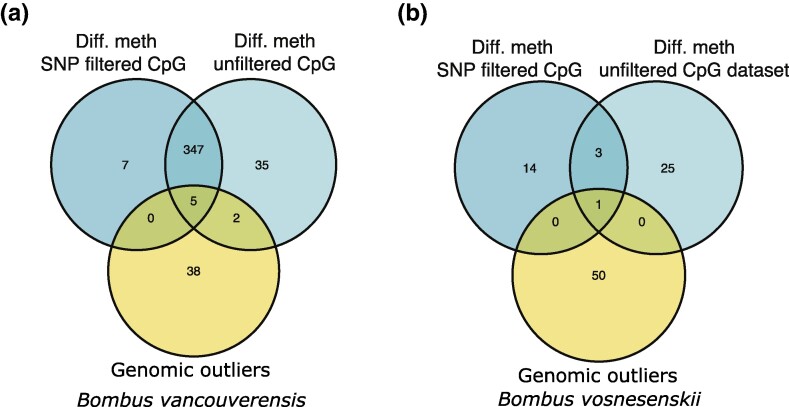
Venn diagrams comparing the number of genes recovered as differentially methylated without or without the SNP filter in a) *B. vancouverensis* or b) *B. vosnesenskii*. All comparisons also include lists of genes with previously identified environmentally associated outlier SNPs (Heraghty et al. 2022, 2023).

When considering the genes containing differentially methylated CpGs, SNP filtering had a relatively small effect in *B. vancouverensis*, with most (*n* = 347 [89.2%]) genes identified as containing differentially methylated sites found in both the SNP filtered and unfiltered datasets (Fig. 5a); 7 and 35 genes with differentially methylated CpGs were unique to the SNP filtered and unfiltered datasets, respectively. SNP filtering had a more substantial effect in *B. vosnesenskii*, although this is likely in part due to the relatively small number of differentially methylated regions detected generally (Fig. 5b); only 3 genes with differentially methylated CpGs were shared by the SNP filtered and unfiltered data sets, and 14 and 25 genes were unique to the SNP filtered and unfiltered datasets, respectively (Fig. 5b).

Gene ontology (GO) analysis for *B. vancouverensis* retained 296 biological process terms after summarization with REVIGO (Supek et al. 2011) using the list of genes containing differentially methylated CpG's identified in both the filtered and unfiltered datasets (*n* = 352). Several key clusters of biological processes appear in the GO results involving terms, such as development (e.g. terms like tissue development [GO:0009880], and animal organ morphogenesis [GO:0009887]), RNA processing (e.g. terms like RNA splicing [GO0008380], mRNA splicing via spliceosome [GO:0000398]), and hypoxia (e.g. terms like response to hypoxia [GO:0001666] and response to oxygen levels [GO:0070482]). Because there were only four genes found in both filtered and unfiltered dataset in *B. vosnesenskii*, instead of summarizing gene function with a GO approach we elected to evaluate the function of each of the 4 genes manually. To do this, we assessed the putative gene function by first determining if there was a homologous gene in *Drosophila melanogaster* using the *blast_rec* function in the orthologr v.0.4.2 R package (Drost et al. 2015) and then searched for available information on FlyBase. Two of the four genes had homologous genes in *D. melanogaster* with one of the genes being LOC117239631 which is homologous to *Dp1* and is involved in processes like mRNA translation (Nelson et al. 2007). LOC117237625 is homologous to *sli* which is involved in several different developmental processes including neuronal and tracheal development (Rothberg et al. 1990; Englund et al. 2002).

## Discussion

We used enzymatic methylation sequencing to evaluate the distribution of methylated CpGs across the genome and variation in methylation across the geographic range of 2 broadly distributed bumble bee species. Consistent with prior results in bumble bees and many other invertebrates (Bewick et al. 2017; Rahman and Lozier 2023), methylation was present at <1% of CpGs in both focal species, with most of the methylated CpGs being in gene bodies, especially in exons. Overall spatial or environmentally associated patterns of differential methylation among populations within the species were not particularly striking and were generally less dramatic than previously examined SNP variation across the landscape in these species, although some patterns were evident. In particular, differences in methylation were more pronounced in *B. vancouverensis* than in *B. vosnesenskii*, including many CpGs significantly associated with elevation that reemphasizes the potential relevance of this spatial–environmental dimension for this species observed in earlier studies of genotypic and phenotypic variation (Pimsler et al. 2020; Lozier et al. 2021; Heraghty et al. 2023, 2022).

General patterns of methylome-wide differentiation across the ranges of both species were generally weak (Figs. 3 and 4). Previous work using genomic SNPs suggests that population structure in *B. vancouverensis* is on a north south gradient that loosely breaks down into a southern population (California) and a northern population (Oregon and Washington) with strong isolation by distance and environment (Jackson et al. 2018; Heraghty et al. 2022). The RDA recovered a somewhat similar pattern, with individuals generally clustering into northern and southern groups, although this clustering is less than observed in the genomic data (Jackson et al. 2018; Heraghty et al. 2022). Signatures of isolation by distance are also smaller than for SNP data in the same *B. vancouverensis* samples (supplementary fig. S1, Supplementary Material online), suggesting that spatial population structuring is not as prevalent in methylation as in genome sequence variation. In *B. vosnesenskii*, a similar set of genomic analyses found little population structure at the range-wide scale (Jackson et al. 2018; Heraghty et al. 2022), which is consistent with the minimal structure also found in the methylation data presented here.

In addition to spatial–environmental predictors of methylation, we also aimed to directly test the hypothesis that genetic background shapes methylation by examining differences in methylation in relation to genetic distance among individuals. Previous work in bumble bees has found a high level of intercolony variation in methylation, suggesting that genetic background may play a role in epigenetic processes (Marshall et al. 2019). Given population genetic structuring, especially in *B. vancouverensis*, such relatedness effects might be expected to extend to the population level and potentially result in “epialleles” that could be targeted by selection, although it remains unclear if such a phenomenon exists in *Bombus* (Marshall et al. 2019). Similar to the isolation-by-distance results (Fig. 3), relatively little variation was explained by the Mantel tests comparing methylation dissimilarity and genomic distance, but relationships were all positive and at least some comparisons were significant. This suggests that there may be some impact of genetic background on methylation at the range-wide scale, but the inherently noisier quantitative methylation data have a great deal of individual-specific variation that may require larger sample sizes for robust conclusions to be drawn. Thus, more work will be necessary to specifically test the ways in which genetic background may facilitate variation in methylation among individuals and populations (Chapelle and Silvestre 2022), as well as to identify optimal data filtering and CpG inclusion strategies for low-methylation species like bumble bees.

At least some methylation variation was associated with environmental variables in each species, however, both methylome-wide (e.g. Fig. 4, Table 2) and at individual CpGs. The CpG-level differential methylation was once again much clearer in *B. vancouverensis* than in *B. vosnesenskii*. Of particular interest was that the largest number of significant CpGs was associated with elevation in *B. vancouverensis*, which is intriguing given prior studies indicating elevation as an important driver of multiple evolutionary processes in this species. For example, high elevation regions are associated with gene flow reductions (Jackson et al. 2018) as well as shifts in body size and wing loading that may benefit flight in challenging high-altitude conditions (Lozier et al. 2021). In addition, genome-wide outlier analysis in *B. vancouverensis* identified genes containing multiple SNPs associated with elevation and may indicate local adaptation, in particular the gene *Mrp4* that is involved in resistance to hypoxia (Heraghty et al. 2023), a key stressor at altitude (Dillon 2006). GO results for differentially methylated CpGs in *B. vancouverensis* also indicate several terms related to hypoxia (e.g. response to hypoxia [GO:0001666] and response to oxygen levels [GO:0070482]). One possible role for the large number of CpGs associated with elevation may thus be a mechanism for regulating gene expression to counteract hypoxic conditions (Harrison et al. 2018). The lack of environmentally associated differential methylation in *B. vosnesenskii* is similar to genomic studies which found few environmentally associated SNPs (Jackson et al 2020, Heraghty et al 2023), which creates an interesting question for *B. vosnesenskii*. Recent models suggest an increase in range and abundance as climate change continues to progress (Soroye et al. 2020; Jackson et al. 2022) and have also suggested that increases in temperature may be the key mechanism underlying these increases. Although there is variation in CT_min_ across the range of this species (Pimsler et al. 2020), there is little evidence suggesting that temperature is strongly shaping molecular variation (Heraghty et al. 2023). Given that CT_min_ may be important in understanding range expansions (e.g. increased temperatures means populations can move further north before experiencing limiting temperatures), more work will be needed to understand the molecular mechanisms may drive variation in this trait.

One of the major objectives of this study was to compare 2 bumble bees species provide insights into how differences in environmental niche (greater specialization to higher elevations in *B. vancouverensis* vs. greater habitat generalism in *B. vosnesenskii*) may influence evolution of DNA methylation variation. The greater level of environmental association observed at CpGs for *B. vancouverensis* relative to *B. vosnesenskii* has also been observed in genomic SNP data (Heraghty et al. 2023, 2022) and morphological data (Lozier et al. 2021), suggesting that to some extent different processes act in parallel, although the strength of such associations clearly varies. It is possible that DNA sequence and methylation may act in concert to achieve adaptive physiologies across the landscape however, the small overlap in genes with differential methylation and environmentally associated SNPs suggests that any possible parallels that emerge across a species range are likely involving different mechanisms. Further, even in *B. vancouverensis*, the spatial and environmental associated population structure in the methylome is weaker than other data types, which together with other studies finding similar relationships between genomic and epigenetic data (e.g. Richards et al. 2012, Sheldon et al. 2018 suggest the ultimate drivers of methylation variation across species ranges may be complex and not easily predicted solely from patterns of genomic variation). However, other studies have identified stronger correlations between genomic background and variation in methylation, including data in bumble bees that found high methylation variation between colonies with colony of origin better explaining individual CpG methylation patterns than caste differences between individuals (Marshall et al. 2019). It may be the case that study design, particularly using wild or laboratory animals and the number of samples representing distinct colonies or populations, may influence the power to detect some methylation patterns compared to other types of variation, and it may be that taxonomic groups with more widespread methylation have greater genetic control over the distribution of this methylation (Chapelle and Silvestre 2022). Understanding the relative contributions of the genetic background and DNA methylation patterns to environmental adaptation in wild populations will likely continue to be an important area of research (Husby 2022).

Regardless of the causes of inter-individual and inter-population variation in DNA methylation, there is not yet a clear consensus on the role of this epigenetic mechanism in the evolution of arthropods, and we still cannot explain how differences in DNA methylation may be ultimately impacting the biology of the focal bumble bee species. Our results did find most of the DNA methylation to be occurring in gene bodies, which is consistent with other arthropods (Bewick et al. 2017; Rahman and Lozier 2023). There are some theories regarding the role of gene body methylation including potential interactions with other epigenetic factors (Glastad et al. 2014, 2015), alternative splicing (Li-Byarlay et al. 2013; Marshall et al. 2019; Lewis et al. 2020), and the seesaw hypothesis (increases/decreases in methylation to drive decreases/increases in gene expression) (Dixon et al. 2018; Dixon and Matz 2022). However, more work will need to be done to understand the evolutionary role of DNA methylation in this lineage, such as the importance of environmental pressures as causal forces in driving flexible methylation variation at the individual level and whether selection can act on “epialleles” to produce more stably inherited and locally adapted methylation patterns (Burggren 2016; Bewick et al. 2017) that influence some other downstream molecular process.

Finally, there are several important caveats to our results. First, methylation patterns are tissue specific with different tissues having different methylation profiles. Given the role of thoracic muscle in flight and thermal regulation (Heinrich 1977), we considered this tissue to be a useful starting point for beginning to assess variation in methylation since these 2 processes are likely linked to the environment (Pimsler et al. 2020; Rahman and Lozier 2023). However, a comprehensive understanding of how methylation varies with the environment will require studying other tissue types. For instance, differential methylation in the fat body might be more likely to be found in genes linked to metabolic functions (Arrese and Soulages 2010). Incorporating data on queens and males in wild populations may also be important, since these may also impact methylation profiles (Yan et al. 2015; Harrison et al. 2022) and these life stages are active at unique times during the season and experience distinct environmental pressures from workers (Woodard 2017). Second, research into methylation patterns of nonmodel organisms in natural settings poses unique challenges compared to controlled experiments, such as noise due to unaccounted for differences in individual environmental exposure and life history, such as age (Yan et al. 2015; Harrison et al. 2022; Renard et al. 2023). Controlling for these differences may require specific experimental design choices, like employing sampling strategies to minimize differences between samples or simply increasing sample size. Alternatively, as done in this study as well as others (Liew et al. 2020; Rahman and Lozier 2023), applying minimum methylation thresholds may help reduce such noise, but sensitivity of results to the method of filtering suggests multiple criteria should be evaluated. Finally, another concern is overall sampling design of methylation studies, including sequencing depth. A recent bisulfite sequencing study between 2 populations from extreme ends of the range in *B. vosnesenskii* found over 2,000 differentially methylated genes (Rahman and Lozier 2023) compared to the <100 found here. However, that study (Rahman and Lozier 2023) followed a case–control design compared to our continuous predictor design and had many times greater sequencing depth (∼76×) than in this study (∼14× for *B. vancouverensis* and ∼22× *B. vosnesenskii*) which together may enhance power to identify subtle differential methylation that might be common in bumble bees (Pozo et al. 2021). For example, Rahman and Lozier (2023) were able to detect many differentially methylated CpGs using a threshold of 10%, which would have been difficult here due to our lower sequencing depth and minimum depth threshold. Thus, although we gain several insights with our current data, higher sequencing effort than might typically be applied to genomic data will be needed to detect many methylation differences, even for methods like Enzymatic Methyl Seq that are less damaging to DNA than methods like bisulfite sequencing.

In conclusion, we provide more evidence that patterns in DNA methylation are relatively conserved across insects based on the overall low number of methylated CpGs identified here and that methylation occurs primarily in exons. Our results also provide some of the first insights into how methylation varies across the landscape in wild populations of bumble bees. We do find some clear patterns, like the association between methylation and elevation in *B. vancouverensis*. This will be a useful starting point for future studies that can more rigorously identify the specific effects different stimuli might have on DNA methylation as well as how these sorts of effects might differ depending on the genomic background. Given the relative lack of population structure in the methylation data, it may be that methylation does not play a major role in local adaptation, although our results do not exclude the possibility that methylation may be in involved in complementary processes like plasticity that could serve to help individuals cope with environmental stresses. We also provide some methodical considerations for future studies that may also be using field caught samples as well as provide some possible solutions. Overall, the results suggest there is indeed variation in methylation across the landscape that has possible biological implications and consequently merits further study.

## Materials and Methods

### Sample Collection and DNA Extraction

Sampling sites were located on a latitudinal range of 37.217° to 48.651°N for *B. vancouverensis* and 36.619° to 45.627°N for *B. vosnesenskii* and covered a wide elevational breadth of 447 to 2,678 m for *B. vancouverensis* and 68 to 2497 m for *B. vosnesenskii* (supplementary table S1, Supplementary Material online). Female workers were collected by net and transferred to vials of 100% ethanol kept on ice in the field, and ultimately stored at −80 °C until DNA extraction. Qiagen DNeasy blood and tissue kits (Hilden, N.R.W., Germany) were used to extract DNA from thoracic muscle tissue. DNA methylation libraries were prepared using the NEB Enzymatic Methyl Seq kit (Ipswich, MA, USA). All samples were spiked with 1 μL of methylated pUC19 control DNA and 1 μL of unmethylated lambda DNA to assess the success of methylation conversion. Samples were sequenced across 2 lanes on an Illumina NovaSeq 600 sequencer (Psomagen, Rockville, MD, USA).

### Bioinformatic Processing

Trim Galore! v0.6.6 (Krueger 2015) was used to trim and remove low quality reads using conservative hard trimming flags due to possible issues with the conversion process that can reduce quality at ends of reads: –q 20 –clip_R1 10 –clip_R2 15 –three_prime_clip_R2 10 –length 50. Trimmed reads were aligned to against the *B. vancouverensis* (NCBI RefSeq ID: GCF_011952275.1) and *B. vosnesenskii* (NCBI RefSeq ID: GCF_011952255.1) genomes (Heraghty et al. 2020) using bwa-meth v0.2.2 (Pedersen et al. 2014). The alignment files were then converted into binary format (BAM) and sorted using SAMtools v1.10 (Li et al. 2009). PCR and optical duplicates were removed using the *markduplicates* command in Picard Tools v2.20.4 (Broad Institute 2019). Methylation was detected using MethyDackel v0.6.1 (https://github.com/dpryan79/MethylDackel) with the –minDepth 6 flag to only consider bases with a sequencing depth of at least 6 reads. We also generated a filtered dataset where putative SNP variant sites were removed bioinformatically within MethylDackel using the –maxVariantFrac 0.50 –minOppositeDepth 3 flags. This filtered dataset was then further filtered by removing SNPs previously detected in recent range-wide whole genome resequencing for both species (Heraghty et al. 2022, 2023) (supplementary data, Supplementary Material). To ensure the possible impact of SNPs on CpG motifs was fully removed, we also removed sites from the MethylDackel output files that were adjacent to each SNP (±1 bp) using the GenomicRanges v1.48.0 R package (Lawrence et al. 2013) in R v4.2.0 (R Core Team 2002). All subsequent analyses were done on both the unfiltered and SNP-filtered datasets.

MethylDackel output files were read into R using the methylKit R package v1.20.0 (Akalin et al. 2012) for further filtering. MethylKit requires samples be assigned to a treatment group, so for the purposes of importing, uniting, and downstream data processing to filter methylations calls for other analyses, *B. vancouverensis* samples were assigned to either a northern or southern group which corresponded to previously identified patterns in population structure using genomic data (Heraghty et al. 2022). *Bombus vosnesenskii* does not have such significant population structure but does exhibit weak isolation by distance that is most evident with latitudinal separation at large scales (Heraghty et al. 2023); we thus elected to split samples into northern and southern groups based on the mean latitude of sampling localities (40.2°N, with samples above this latitude being classified as “northern” and samples below that latitude being classified as “southern”) to account for the minimal population structure when classifying the imported datasets into methylKit. Note that these sample groups are only used for importing, joining, and filtering MethylDackel sample outputs in methylKit, not for any statistical analysis, so these assigned groups should not impact downstream analyses. For each species, methylKit was utilized to remove any sites with unusually high coverage (>99% percentile of coverage) by using the *filterByCoverage* command and to normalize read counts using the *normalizeCoverage* function (median method). Finally, the data for each sample were combined into a single methylBase object using the *unite* command, which only retained CpG sites found in 70% of samples in a given group to ensure that included sites were represented across the spatial range of the species (e.g. a CpG position had to be sequenced in at least 70% of individuals in northern and southern *B. vancouverensis* groups). This produced a file for all sequenced CpGs (methylated and unmethylated).

### Characterizing the Distribution of Methylated CpGs in the Genome

The united methylKit output file for each dataset for each species was transformed into a percent methylation matrix using the *percMethylation* command in methylKit and used to calculate overall percent methylation for each species. In addition to evaluating the all CpG data set, to assess the distribution of methylated CpGs in the genome of each species, the percent methylation matrix was also filtered to include only CpGs with ≥30% methylation on average across all samples (hereafter referred to as the “Meth30 dataset”). This procedure thus focused on moderately to highly methylated sites in the genome and reduced noise in differential methylation analyses from CpG sites harboring low levels of methylation, which represent most CpGs across bumble bee genomes (Marshall et al. 2019; Rahman and Lozier 2023). Analysis of the Meth30 dataset provides insight into general patterns of methylation across the range of both species. The threshold of 30% was selected after manual inspection of the data, which suggested this value was useful in reducing statistical noise from low methylation sites that may reflect sequencing error or occasional poor enzymatic conversion.

We also compared results to a dataset of “highly variable sites” generated using a threshold of two standard deviations (SD > 2) of percent methylation (hereafter referred to as the “HVar dataset”). The HVar dataset represents CpGs that have relatively higher variation in percent methylation across all samples and may be more likely to reveal differential responses to environmental or spatial variables (e.g. similar to a minor allele frequency in SNP-based outlier analyses; Rahman and Lozier 2023). Although this removed most low-variability CpGs, due to the low overall methylation proportion across the genome, even after the SD filter we noted some methylated sites were still only observed in 1 or 2 samples with low percent methylation values. We thus enforced a secondary requirement for the HVar dataset that all CpGs had to be methylated in ≥4 individuals. This eliminated low-frequency methylated sites or those that might be erroneously identified as variable based on a single low methylation site in a single individual which could arise from an error in enzymatic conversion or sequencing, with this threshold selected to strike a balance between removal of too many CpG sites and retention of sites consistently methylated in multiple samples. At the end of these processing steps, we had 6 data sets for each species: SNP-filtered/All-CpGs, unfiltered/All-CpGs, SNP-filtered/Meth30, unfiltered/Meth30, SNP-filtered/HVar, and unfiltered/HVar.

To evaluate how CpGs were distributed across the genome, general feature format (.gff) files were generated from the gene transfer format (.gtf) files on NCBI RefSeq (Heraghty et al. 2020) for each genomic feature of interest (exon, intron, start codon, stop codon, 3′ untranslated region [UTR], and 5′ UTR). We used AGAT v.0.7.0 to retain features for only the longest transcript (Dainat 2023). The *intersect* command from BEDTools v.2.30.0 (Quinlan and Hall 2010) was used to produce feature-specific annotation for individual CpGs in each dataset. CpG sites that did not intersect any genomic features were classified as intergenic. Chi-square tests were performed using the *chisq.test* function in R to compare the distributions all CpGs between different feature categories.

### Spatially and Environmentally Associated Differential Methylation

#### Differential Methylation

In previous genome-wide SNP studies in *B. vancouverensis* and *B. vosnesenskii*, we identified significant effects of spatial and environmental variation in both species (Jackson et al. 2020; Heraghty et al. 2023, 2022). To assess the role of geography and environmental variation in shaping methylation patterns, we first tested for differential methylation associated with several spatial and environmental variables using the MethylSig v.1.6.0 R package (Park et al. 2014). The raw BedGraph output files generated by MethylDackel were read into a BS-seq object and filtered to retain only CpG sites found in the HVar dataset described above. Predictor variables were then selected to represent spatial and bioclimatic variation across the sampled range for both species. We used latitude to represent spatial variation, which represents the major geographic axis of sampling in this study and is a major spatial predictor of SNP variation (Jackson et al. 2018). For environmental variables, we used the same variables selected in prior studies of SNP variation (Heraghty et al. 2022, 2023), in which bioclimatic environmental variables (BioClim variables) were selected from the WorldClim2 dataset (0.5 arc-minute resolution) (Fick and Hijmans 2017) using a variable reduction strategy to minimize correlations. For *B. vancouverensis*, these included BIO1, Mean Diurnal Range (BIO2), BIO3, and BIO12. For *B. vosnesenskii*, BIO1, BIO3, and BIO12 were selected. For both species, elevation was also included in the analysis. Although elevation is often correlated with other bioclimatic variables, it can capture some unique environmental variation of interest (such as reduced air pressure and lower oxygen levels (Dillon 2006)) and prior research in bumble bees indicates elevation may produce unique genomic signatures not observed in other variables (Sun et al. 2020; Liu et al. 2020b; Heraghty et al. 2022).

We ran differential methylation tests specifying each spatial and environmental variable as a numerical covariate using the *diff_dss_fit* and *diff_dss_test* functions in MethylSig. The resultant *P*-values were transformed into *q*-values to account for multiple testing using the qvalue v2.26.0 R package (Storey et al. 2022). A given CpG site was considered to be significantly differentially methylated at a false discovery rate corrected threshold of *q* ≤ 0.05. Individual CpGs with statistically significant differential methylation were intersected with the previously generated genomic feature files to identify genes harboring differentially methylated sites. To identify general trends in function of differentially methylated genes, we conducted a GO enrichment analysis via the *go_enrich* function in the GOfuncR v.1.16.0 R package. Species-specific GO terms were obtained from the Hymenoptera genome database (Walsh et al. 2021), with differentially methylated genes being considered as candidate genes and all other genes specified as the background set. GO terms with *P*-value < 0.01 were retained and subsequently summarized with REVIGO web server (Supek et al. 2011) using the “medium (0.7)” stringency filter. Differentially methylated genes were also compared against a list of genes (*n* = 45 for *B. vancouverensis* and *n* = 51 for *B. vosnesenskii*) previously identified as being associated with environmental variables based on whole genome SNP data (Heraghty et al. 2022, 2023).

#### Methylome-wide Associations with Space, Environment, and Genetic Variation

As a second approach to detect general differentiation in methylation with spatial separation or environment (as opposed to differential methylation at individual CpG sites), we conducted methylome-wide analyses of isolation by distance and environment. We generated methylation distances among samples for each species by first imputing missing data in both the Meth30 and HVar datasets to facilitate downstream analyses that required datasets with no missing data. Imputation was conducted using the *imputePCA* function of the R package missMDA v.1.19 (Josse and Husson 2016), which uses an iterative PCA algorithm and has been shown to perform well on methylation datasets (Lena et al. 2020). We then used the imputed data to generate a pairwise matrix of Gower's dissimilarity among individuals using the *daisy* function in the R package cluster v.2.1.4 (Maechler et al. 2022). Gower's dissimilarity is a flexible metric for assessing sample differences, with 0 being identical and 1 being completely different (Gower 1987; Lin et al. 2015; Koch et al. 2016). Pairwise geographic distances were calculated between each individual and each sampling site using the *distm* function in the geosphere v.1.5-18 R package (Hijmans 2022). Mantel tests were used to test for significant correlations between geographic distance and methylation dissimilarity using the *mantel* function with 1,000 permutations from the vegan v.2.6-4 R package (Oksanen et al. 2022).

Given previously noted connections between an individual's genetic background and CpG methylation (Marshall et al. 2019; Chapelle and Silvestre 2022), we also identified SNPs from the methyl-seq data to directly compare genetic and methylation differences among individuals. SNPs were called from the trimmed sequencing reads using BISCUIT v.1.2.0 (Zhou 2024), following the author's suggested workflow (https://huishenlab.github.io/biscuit/). First, trimmed reads were aligned to the reference genome via the BISCUIT align command, SAMBLASTER v.0.1.26 was used to sort and mark duplications, and then the SAMtools *view* command was used to create final alignment files in BAM. Second, SNPs were called using the *pileup* command in BISCUIT and filtered using BCFtools v.1.17 (Li et al. 2009) to retain biallelic sites with less than 25% missing data and a base quality score *Q* > 30. The *gl.dist.ind* function from the dartR v.2.7.2 R package (Gruber et al. 2018) was used to generate genetic distances between individuals. As a method of quality control we also performed a PCA on a subset of SNPs with no missing data (2,969 SNPs for *B. vancouverensis* and 3,200 SNPs for *B. vosnesenskii*) using the *gl.pcoa* command in the dartR package. We used Mantel tests to test the significance of the association between the individual-level genomic distance and Gower's dissimilarity matrices as described above. We also conducted a Mantel test using the individual genomic distance and geographic distance matrices to confirm prior results regarding genetic isolation by distance for each species (Heraghty et al. 2022, 2023).

Finally, we conducted partial redundancy analysis (pRDA) to identify patterns of methylome-wide variation that could be explained by environmental variables, geographic variables, and population structure. This approach is useful in obtaining both the total amount of variation accounted for by all explanatory variables as well as each for different subsets of environmental (BioClim variables and elevation, referred to as env) or geographic (latitude and longitude, referred to as geo) variables (Capblancq and Forester 2021). To account for population structure, we included the first principal component (PC) axis from the PCA analysis of the BISCUIT SNP data (referred to as pop). The model run using all explanatory variables is referred to as the full model (F ∼ env + geo + pop) and accounts for the total amount of variation that can be explained by all variables. We then ran three partial models to account for the individual effects of environment (F ∼ env | [geo + pop]), geography (F ∼ geo | [env + pop]), and population structure (F ∼ pop | [env + geo]) respectively while factoring in the effect of the other conditioned variables (e.g. the geography models asses the amount of variation only explained by geographic variables).

## Supplementary Material

evae207_Supplementary_Data

## Data Availability

All raw sequencing data are available on NCBI under BioProject PRJNA998862. The list of genes with environmentally associated SNPs for both species, the.vcf file of SNPs called by the biscuit pipeline after filtering, files containing the list of previously identified SNPs used in filtering for both species, and the methylKit objects containing all CpG (methylated and unmethylated) sites can be found in the supplementary data, Supplementary Material online at FigShare. The code used for this experiment can also be found in the supplementary data, Supplementary Material online at FigShare.

## References

[evae207-B1] Ahrens CW , Rymer PD, Stow A, Bragg J, Dillon S, Umbers KDL, Dudaniec RY. The search for loci under selection: trends, biases and progress. Mol Ecol. 2018:27(6):1342–1356. 10.1111/mec.14549. 29524276

[evae207-B2] Akalin A , Kormaksson M, Li S, Garrett-Bakelman FE, Figueroa ME, Melnick A, Mason CE. MethylKit: a comprehensive R package for the analysis of genome-wide DNA methylation profiles. Genome Biol. 2012:13(10):R87. 10.1186/gb-2012-13-10-R87. 23034086 PMC3491415

[evae207-B3] Arrese EL , Soulages JL. Insect fat body: energy, metabolism, and regulation. Annu Rev Entomol. 2010:55(1):207–225. 10.1146/annurev-ento-112408-085356. 19725772 PMC3075550

[evae207-B4] Bain SA , Marshall H, de la Filia AG, Laetsch DR, Husnik F, Ross L. Sex-specific expression and DNA methylation in a species with extreme sexual dimorphism and paternal genome elimination. Mol Ecol. 2021:30(22):5687–5703. 10.1111/mec.15842. 33629415

[evae207-B5] Bebane PSA , Hunt BJ, Pegoraro M, Jones ARC, Marshall H, Rosato E, Mallon EB. The effects of the neonicotinoid imidacloprid on gene expression and DNA methylation in the buff-tailed bumblebee *Bombus terrestris*. Proc R Soc Lond B Biol Sci. 2019:286:20190718. 10.1098/rspb.2019.0718. PMC6599982 31213186

[evae207-B6] Bewick AJ , Vogel KJ, Moore AJ, Schmitz RJ. Evolution of DNA methylation across insects. Mol Biol Evol. 2017:34(3):654–665. 10.1093/molbev/msw264. 28025279 PMC5400375

[evae207-B7] Bonasio R , Li Q, Lian J, Mutti NS, Jin L, Zhao H, Zhang P, Wen P, Xiang H, Ding Y, et al Genome-wide and caste-specific DNA methylomes of the ants *Camponotus floridanus* and *Harpegnathos saltator*. Curr Biol. 2012:22(19):1755–1764. 10.1016/j.cub.2012.07.042. 22885060 PMC3498763

[evae207-B8] Broad Institute. 2019. https://broadinstitute.github.io/picard/.

[evae207-B9] Burggren W . Epigenetic inheritance and its role in evolutionary biology: re-evaluation and new perspectives. Biology (Basel). 2016:5(2):1–22. 10.3390/biology5020024. PMC4929538 27231949

[evae207-B10] Cameron SA , Lozier JD, Strange JP, Koch JB, Cordes N, Solter LF, Griswold TL. Patterns of widespread decline in North American bumble bees. Proc Natl Acad Sci USA. 2011:108(2):662–667. 10.1073/pnas.1014743108. 21199943 PMC3021065

[evae207-B11] Cameron SA , Sadd BM. Global trends in bumble bee health. Annu Rev Entomol. 2020:65(1):209–232. 10.1146/annurev-ento-011118-111847. 31610137

[evae207-B12] Capblancq T , Forester BR. Redundancy analysis: a Swiss army knife for landscape genomics. Methods Ecol Evol. 2021:12(12):2298–2309. 10.1111/2041-210X.13722.

[evae207-B13] Carvalho CFD . Epigenetic effects of climate change on insects. Curr Opin Insect Sci. 2023:57:1–7. 10.1016/j.cois.2023.101029. 37028647

[evae207-B14] Cayuela H , Dorant Y, Mérot C, Laporte M, Normandeau E, Gagnon-Harvey S, Clément M, Sirois P, Bernatchez L. Thermal adaptation rather than demographic history drives genetic structure inferred by copy number variants in a marine fish. Mol Ecol. 2021:30(7):1–18. 10.1111/mec.15835. 33565147

[evae207-B15] Chano V , Domínguez-Flores T, Hidalgo-Galvez MD, Rodríguez-Calcerrada J, Pérez-Ramos IM. Epigenetic responses of hare barley (*Hordeum murinum subsp. leporinum*) to climate change: an experimental, trait-based approach. Heredity (Edinb). 2021:126(5):748–762. 10.1038/s41437-021-00415-y. 33608652 PMC8102545

[evae207-B16] Chapelle V , Silvestre F. Population epigenetics: the extent of DNA methylation variation in wild animal populations. Epigenomes. 2022:6(4):1–25. 10.3390/epigenomes6040031. PMC9589984 36278677

[evae207-B17] Dainat J . AGAT: another GFF analysis toolkit to handle annotations in any GTF/GFF format. (version v0.7.0). Zenodo. 2023. 10.5281/zenodo.3552717.

[evae207-B18] Dillon ME . Into thin air: physiology and evolution of alpine insects. Integr Comp Biol. 2006:46(1):49–61. 10.1093/icb/icj007. 21672722

[evae207-B19] Dillon ME , Lozier JD. Adaptation to the abiotic environment in insects: the influence of variability on ecophysiology and evolutionary genomics. Curr Opin Insect Sci. 2019:36:131–139. 10.1016/j.cois.2019.09.003. 31698151

[evae207-B20] Dixon G , Liao Y, Bay LK, Matz MV. Role of gene body methylation in acclimatization and adaptation in a basal metazoan. Proc Natl Acad Sci USA. 2018:115(52):13342–13346. 10.1073/pnas.1813749115. 30530646 PMC6310852

[evae207-B21] Dixon G , Matz M. Changes in gene body methylation do not correlate with changes in gene expression in Anthozoa or Hexapoda. BMC Genomics. 2022:23(1):234. 10.1186/s12864-022-08474-z. 35337260 PMC8957121

[evae207-B22] Dorant Y , Laporte M, Rougemont Q, Cayuela H, Rochette R, Bernatchez L. Landscape genomics of the American lobster (*Homarus americanus*). Mol Ecol. 2022:31:1–19. 10.1111/mec.16653. 35960266 PMC9805075

[evae207-B23] Drost HG , Gabel A, Grosse I, Quint M. Evidence for active maintenance of phylotranscriptomic hourglass patterns in animal and plant embryogenesis. Mol Biol Evol. 2015:32(5):1221–1231. 10.1093/molbev/msv012. 25631928 PMC4408408

[evae207-B24] Englund C , Steneberg P, Falileeva L, Xylourgidis N, Samakovlis C. Attractive and repulsive functions of slit are mediated by different receptors in the *Drosophila* trachea. Development. 2002:129(21):4941–4951. 10.1242/dev.129.21.4941. 12397103

[evae207-B25] Fick SE , Hijmans RJ. WorldClim 2: new 1-km spatial resolution climate surfaces for global land areas. Int J Climatol. 2017:37(12):4302–4315. 10.1002/joc.5086.

[evae207-B26] Flores KB , Wolschin F, Amdam GV. The role of methylation in environmental adapatation. Integr Comp Bio. 2013:53(2):359–372. 10.1093/icb/ict019. 23620251 PMC3710460

[evae207-B27] Gao Y , Chen Y, Li S, Huang X, Hu J, Bock DG, MacIsaac HJ, Zhan A. Complementary genomic and epigenomic adaptation to environmental heterogeneity. Mol Ecol. 2022:31:1–15. 10.1111/mec.16500. 35560847

[evae207-B28] Glastad KM , Hunt BG, Goodisman MA. Evolutionary insights into DNA methylation in insects. Curr Opin Insect Sci. 2014:1:25–30. 10.1016/j.cois.2014.04.001. 32846726

[evae207-B29] Glastad KM , Hunt BG, Goodisman MAD. DNA methylation and chromatin organization in insects: insights from the ant *Camponotus floridanus*. Genome Biol Evol. 2015:7(4):931–942. 10.1093/gbe/evv039. 25724207 PMC4419788

[evae207-B30] Gower JC . A general coefficient of similarity and some of its properties. Proc IEEE Ultrason Symp. 1987:27:837–842. 10.1109/ultsym.1987.199076.

[evae207-B31] Greenleaf SS , Kremen C. Wild bee species increase tomato production and respond differently to surrounding land use in Northern California. Biol Conserv. 2006:133(1):81–87. 10.1016/j.biocon.2006.05.025.

[evae207-B32] Gruber B , Unmack PJ, Berry OF, Georges A. Dartr: an r package to facilitate analysis of SNP data generated from reduced representation genome sequencing. Mol Ecol Resour. 2018:18(3):691–699. 10.1111/1755-0998.12745. 29266847

[evae207-B33] Gupta A , Nair S. Heritable epigenomic modifications influence stress resilience and rapid adaptations in the brown planthopper (*Nilaparvata lugens*). Int J Mol Sci. 2022:23(15):1–30. 10.3390/ijms23158728. PMC9368798 35955860

[evae207-B34] Harney E , Paterson S, Collin H, Chan BHK, Bennett D, Plaistow SJ. Pollution induces epigenetic effects that are stably transmitted across multiple generations. Evol Lett. 2022:6(2):1–18. 10.1002/evl3.273. PMC8966472 35386832

[evae207-B35] Harrison MC , Dohmen E, George S, Sillam-Dussès D, Séité S, Vasseur-Cognet M. Complex regulatory role of DNA methylation in caste- and age-specific expression of a termite. Open Biol. 2022:12(7):220047. 10.1098/rsob.220047. 35857972 PMC9256085

[evae207-B36] Harrison JF , Greenlee KJ, Verberk WCEP. Functional hypoxia in insects: definition, assessment, and consequences for physiology, ecology, and evolution. Annu Rev Entomol. 2018:63(1):303–325. 10.1146/annurev-ento-020117-043145. 28992421

[evae207-B37] Hartke J , Waldvogel A-M, Sprenger PP, Schmitt T, Menzel F, Pfenninger M, Feldmeyer B. Little parallelism in genomic signatures of local adaptation in two sympatric, cryptic sister species. J Evol Biol. 2021:34(6):937–952. 10.1111/jeb.13742. 33200473

[evae207-B38] Heinrich B . The physiology of exercise in the bumblebee. Am Sci. 1977:65:455–465. http://www.jstor.org/stable/27847968.

[evae207-B39] Heraghty SD , Jackson JM, Lozier JD. Whole genome analyses reveal weak signatures of population structure and environmentally associated local adaptation in an important North American pollinator, the bumble bee *Bombus vosnesenskii*. Mol Ecol. 2023:20:1–19. 10.1111/mec.17125. 37702957

[evae207-B40] Heraghty SD , Rahman SR, Jackson JM, Lozier JD. Whole genome sequencing reveals the structure of environment-associated divergence in a broadly distributed montane bumble bee, *Bombus vancouverensis*. Insect Syst Divers. 2022:6:1–17. 10.1093/isd/ixac025.

[evae207-B41] Heraghty SD , Sutton JM, Pimsler ML, Fierst JL, Strange JP, Lozier JD. De novo genome assemblies for three North American bumble bee species: *Bombus bifarius*, *Bombus vancouverensis*, and *Bombus vosnesenskii*. G3 2020:10:2585–2592. 10.1534/g3.120.401437. 32586847 PMC7407468

[evae207-B42] Hijmans R . _geosphere: spherical trigonometry_. R package version 1.5-18. 2022. https://CRAN.R-project.org/package=geosphere.

[evae207-B43] Husby A . Wild epigenetics: insights from epigenetic studies on natural populations. Proc Biol Sci. 2022:289:1–9. 10.1098/rspb.2021.1633. PMC8826306 35135348

[evae207-B44] Jackson HM , Johnson SA, Morandin LA, Richardson LL, Guzman LM, M'Gonigle LK. Climate change winners and losers among North American bumblebees. Biol Lett. 2022:18:20210551. 10.1098/rsbl.2021.0551. 35728617 PMC9213113

[evae207-B45] Jackson JM , Pimsler ML, Oyen KJ, Koch-Uhuad JB, Herndon JD, Strange JP, Dillon ME, Lozier JD. Distance, elevation and environment as drivers of diversity and divergence in bumble bees across latitude and altitude. Mol Ecol. 2018:27:2926–2942. 10.1111/mec.14735. 29862596

[evae207-B46] Jackson JM , Pimsler ML, Oyen KJ, Strange JP, Dillon ME, Lozier JD. Local adaptation across a complex bioclimatic landscape in two montane bumble bee species. Mol Ecol. 2020:29:920–939. 10.1111/mec.15376. 32031739

[evae207-B47] Josse J , Husson F. missMDA: a package for handling missing values in multivariate data analysis. J Stat Softw. 2016:70(1):1–31. 10.18637/jss.v070.i01.

[evae207-B48] Kent CF , Dey A, Patel H, Tsvetkov N, Tiwari T, MacPhail VJ, Gobeil Y, Harpur BA, Gurtowski J, Schatz MC, et al Conservation genomics of the declining North American bumblebee *Bombus terricola* reveals inbreeding and selection on immune genes. Front Genet. 2018:9:1–12. 10.3389/fgene.2018.00316. 30147708 PMC6095975

[evae207-B49] Koch IJ , Clark MM, Thompson MJ, Deere-Machemer KA, Wang J, Duarte L, Gnanadesikan GE, McCoy EL, Rubbi L, Stahler DR, et al The concerted impact of domestication and transposon insertions on methylation patterns between dogs and gray wolves. Mol Ecol. 2016:25:1838–1855. 10.5061/dryad.q2g6h. 27112634 PMC4849173

[evae207-B50] Koch J , Strange J, Williams P. Bumble bees of the Western United States. Vol. 143. USDA Forest Service Research Notes; 2012. p. 443–449. 10.1603/0022-0493-99.2.443.

[evae207-B51] Krueger F . 2015. Trim galore. doi:10.5281/zenodo.7598955.

[evae207-B52] Lawrence M , Huber W, Pagès H, Aboyoun P, Carlson M, Gentleman R, Morgan MT, Carey VJ. Software for computing and annotating genomic ranges. PLoS Comput Biol. 2013:9:1–10. 10.1371/journal.pcbi.1003118. PMC3738458 23950696

[evae207-B53] Lena PD , Sala C, Prodi A, Nardini C. Methylation data imputation performances under different representations and missingness patterns. BMC Bioinformatics. 2020:21:1–22. 10.1186/s12859-020-03592-5. 32600298 PMC7325236

[evae207-B54] Lewis SH , Ross L, Bain SA, Pahita E, Smith SA, Cordaux R, Miska EA, Lenhard B, Jiggins FM, Sarkies P. Widespread conservation and lineage-specific diversification of genome-wide DNA methylation patterns across arthropods. PLoS Genet. 2020:16:e1008864. 10.1371/journal.pgen.1008864. 32584820 PMC7343188

[evae207-B55] Li-Byarlay H , Boncristiani H, Howell G, Herman J, Clark L, Strand MK, Tarpy D, Rueppell O. Transcriptomic and epigenomic dynamics of honey bees in response to lethal viral infection. Front Genet. 2020:11:566320. 10.3389/fgene.2020.566320. 33101388 PMC7546774

[evae207-B56] Li-Byarlay H , Li Y, Stroud H, Feng S, Newman TC, Kaneda M, Hou KK, Worley KC, Elsik CG, Wickline SA, et al RNA interference knockdown of DNA methyltransferase 3 affects gene alternative splicing in the honey bee. Proc Natl Acad Sci USA. 2013:110:12750–12755. 10.1073/pnas.1310735110. 23852726 PMC3732956

[evae207-B57] Li H , Handsaker B, Wysoker A, Fennell T, Ruan J, Homer N, Marth G, Abecasis G, Durbin R. The sequence alignment/map format and SAMtools. Bioinformatics. 2009:25:2078–2079. 10.1093/bioinformatics/btp352. 19505943 PMC2723002

[evae207-B58] Li B , Hou L, Zhu D, Xu X, An S, Wang X. Identification and caste-dependent expression patterns of DNA methylation associated genes in *Bombus terrestris*. Sci Rep. 2018:8:1–10. 10.1038/s41598-018-20831-1. 29402971 PMC5799256

[evae207-B59] Li E , Zhang Y. DNA methylation in mammals. Cold Spring Harb Perspect Biol. 2014:6:a019133. 10.1101/cshperspect.a019133. 24789823 PMC3996472

[evae207-B60] Liang C , Liu D, Song P, Zhou Y, Yu H, Sun G, Ma X, Yan J. Transcriptomic analyses suggest the adaptation of bumblebees to high altitudes. Insects. 2022:13:1–14. 10.3390/insects13121173. PMC9783775 36555083

[evae207-B61] Liew YJ , Howells EJ, Wang X, Michell CT, Burt JA, Idaghdour Y, Aranda M. Intergenerational epigenetic inheritance in reef-building corals. Nat Clim Chang. 2020:10:254–259. 10.1038/s41558-019-0687-2.

[evae207-B62] Lin IH , Chen D-T, Chang Y-F, Lee Y-L, Su C-H, Cheng C, Tsai Y-C, Ng S-C, Chen H-T, Lee M-C, et al Hierarchical clustering of breast cancer methylomes revealed differentially methylated and expressed breast cancer genes. PLoS One. 2015:10:e0118453. doi: 10.1371/journal.pone.0118453. 25706888 PMC4338251

[evae207-B63] Liu Y , Jin H, Naeem M, An J. Comparative transcriptome analysis reveals regulatory genes involved in cold tolerance and hypoxic adaptation of high-altitude Tibetan bumblebees. Apidologie. 2020a:51:1166–1181. 10.1007/s13592-020-00795-w.

[evae207-B64] Liu Y , Zhao H, Luo Q, Yang Y, Zhang G, Zhou Z, Naeem M, An J. De novo transcriptomic and metabolomic analyses reveal the ecological adaptation of high-altitude *Bombus pyrosoma*. Insects. 2020b:11:1–14. 10.3390/insects11090631. PMC7563474 32937786

[evae207-B65] Lockett GA , Almond EJ, Huggins TJ, Parker JD, Bourke AFG. Gene expression differences in relation to age and social environment in queen and worker bumble bees. Exp Gerontol. 2016:77:52–61. 10.1016/j.exger.2016.02.007. 26883339

[evae207-B66] Lozier JD , Parsons ZM, Rachoki L, Jackson JM, Pimsler ML, Oyen KJ, Strange J, Dillon ME. Divergence in body mass, wing loading, and population structure reveals species-specific and potentially adaptive trait variation across elevations in montane bumble bees. Insect Syst Divers. 2021:5:1–15. 10.1093/isd/ixab012.

[evae207-B67] Maechler Martin , Rousseeuw P, Struyf A, Hubert M, Hornik K. 2022. cluster: Cluster Analysis Basics and Extensions. R package version 2.1.4. https://CRAN.R-project.org/package=cluster.

[evae207-B68] Marshall H , Lonsdale ZN, Mallon EB. Methylation and gene expression differences between reproductive and sterile bumblebee workers. Evol Lett. 2019:3:485–499. 10.1002/evl3.129. 31636941 PMC6791180

[evae207-B69] Marshall H , Nicholas MT, van Zweden JS, Wäckers F, Ross L, Wenseleers T, Mallon EB. DNA methylation is associated with codon degeneracy in a species of bumblebee. Heredity (Edinb). 2023:130:1–8. 10.1038/s41437-023-00591-z. 36658299 PMC10076500

[evae207-B70] Mccaw BA , Stevenson TJ, Lancaster LT. Integrative and comparative biology epigenetic responses to temperature and climate. Integr Comp Biol. 2020:60:1469–1480. 10.1093/icb/icaa049. 32470117

[evae207-B71] Nakamura S , Yamazaki J, Matsumoto N, Inoue-Murayama M, Qi H, Yamanaka M, Nakanishi M, Yanagawa Y, Sashika M, Tsubota T, et al Age estimation based on blood DNA methylation levels in brown bears. Mol Ecol Resour. 2023:23:1211–1225. 10.1111/1755-0998.13788. 36951099

[evae207-B72] Nelson MR , Luo H, Vari HK, Cox BJ, Simmonds AJ, Krause HM, Lipshitz HD, Smibert CA. A multiprotein complex that mediates translational enhancement in *Drosophila*. J Biol Chem. 2007:282:34031–34038. 10.1074/jbc.M706363200. 17890223

[evae207-B73] Oksanen J , Simpson GL, Blanchet FG, Kindt R, Legendre P, Minchin PR, O'Hara RB, Solymos P, Stevens MHH, Szoecs E, et al vegan: community ecology package. R package version 2.6-4. https://CRAN.R-project.org/package=vegan 2022.

[evae207-B74] Park Y , Figueroa ME, Rozek LS, Sartor MA. MethylSig: a whole genome DNA methylation analysis pipeline. Bioinformatics. 2014:30:2414–2422. 10.1093/bioinformatics/btu339. 24836530 PMC4147891

[evae207-B75] Pedersen BS , Eyring K, De S, Yang IV, Schwartz DA. Fast and accurate alignment of long bisulfite-seq reads. arXiv:1401.1129. 2014:1–2. 10.48550/arXiv.1401.1129, preprint: not peer reviewed.

[evae207-B76] Pimsler ML , Oyen KJ, Herndon JD, Jackson JM, Strange JP, Dillon ME, Lozier JD. Biogeographic parallels in thermal tolerance and gene expression variation under temperature stress in a widespread bumble bee. Sci Rep. 2020:10:1–11. 10.1038/s41598-020-73391-8. 33051510 PMC7553916

[evae207-B77] Pozo MI , Hunt BJ, Van Kemenade G, Guerra-Sanz JM, Wäckers F, Mallon EB, Jacquemyn H. The effect of DNA methylation on bumblebee colony development. BMC Genomics. 2021:22:73–11. 10.1186/s12864-021-07371-1. 33482723 PMC7821684

[evae207-B78] Quinlan AR , Hall IM. BEDTools: a flexible suite of utilities for comparing genomic features. Bioinformatics. 2010:26:841–842. 10.1093/bioinformatics/btq033. 20110278 PMC2832824

[evae207-B79] Rahbek C , Borregaard MK, Antonelli A, Colwell RK, Holt BG, Nogues-Bravo D, Rasmussen CMØ, Richardson K, Rosing MT, Whittaker RJ, et al Building mountain biodiversity: geological and evolutionary processes. Science (1979). 2019a:365:1114–1119. 10.1126/science.aax0151. 31515384

[evae207-B80] Rahbek C , Borregaard MK, Colwell RK, Dalsgaard B, Holt BG, Morueta-Holme N, Nogues-Bravo D, Whittaker RJ, Fjeldså J. Humboldt's enigma: what causes global patterns of mountain biodiversity? Science (1979). 2019b:365:1108–1113. 10.1126/science.aax0149. 31515383

[evae207-B81] Rahman SR , Lozier JD. Constitutive and variable patterns of genome-wide DNA methylation in populations from spatial-environmental range extremes of the bumble bee. Sci Rep. 2023:13:1–45. doi: 10.1038/s41598-023-41896-7. 37689750 PMC10492822

[evae207-B82] R Core Team . 2022. R: A language and environment for statistical computing. Vienna, Austria: R Foundation for Statistical Computing. https://www.R-project.org/.

[evae207-B83] Renard T , Martinet B, De Souza Araujo N, Aron S. DNA methylation extends lifespan in the bumblebee Bombus terrestris. Proc R Soc B. 2023:290(2012):1–10. 10.1098/rspb.2023.2093. PMC10697797 38052245

[evae207-B84] Richards CL , Schrey AW, Pigliucci M. Invasion of diverse habitats by few Japanese knotweed genotypes is correlated with epigenetic differentiation. Ecol Lett. 2012:15:1016–1025. 10.1111/j.1461-0248.2012.01824.x. 22731923

[evae207-B85] Rodríguez-Casariego JA , Mercado-Molina AE, Garcia-Souto D, Ortiz-Rivera IM, Lopes C, Baums IB, Sabat AM, Eirin-Lopez JM. Genome-wide DNA methylation analysis reveals a conserved epigenetic response to seasonal environmental variation in the staghorn coral *Acropora cervicornis*. Front Mar Sci. 2020:7:1–17. 10.3389/fmars.2020.560424.32802822

[evae207-B86] Rothberg JM , Jacobs JR, Goodman CS, Artavanis-Tsakonas S. slit: an extracellular protein necessary for development of midline glia and commissural axon pathways contains both EGF and LRR domains. Genes Dev. 1990:4:2169–2187. 10.1101/gad.4.12a.2169. 2176636

[evae207-B87] Sharif J , Endo TA, Toyoda T, Koseki H. Divergence of CpG island promoters: a consequence or cause of evolution? Dev Growth Differ. 2010:52:545–554. 10.1111/j.1440-169X.2010.01193.x. 20646027

[evae207-B88] Sheldon EL , Schrey A, Andrew SC, Ragsdale A, Griffith SC. Epigenetic and genetic variation among three separate introductions of the house sparrow (*Passer domesticus*) into Australia. R Soc Open Sci. 2018:5:172185. 10.1098/rsos.172185. 29765671 PMC5936936

[evae207-B89] Smith ZD , Meissner A. DNA methylation: roles in mammalian development. Nat Rev Genet. 2013:14:204–220. 10.1038/nrg3354. 23400093

[evae207-B90] Soroye P , Newbold T, Kerr J. Climate change contributes to widespread declines among bumble bees across continents. Science (1979). 2020:367:685–688. 10.1126/science.aax8591. 32029628

[evae207-B91] Storey JD , Bass AJ, Dabney A, Robinson D, Warnes G. 2022. qvalue: Q-value estimation for false discovery rate control. R package version 2.26.0. http://github.com/jdstorey/qvalue.

[evae207-B92] Storfer A , Patton A, Fraik AK. Navigating the interface between landscape genetics and landscape genomics. Front Genet. 2018:9:1–14. 10.3389/fgene.2018.00068. 29593776 PMC5859105

[evae207-B93] Strange JP . *Bombus huntii*, *Bombus impatiens*, and *Bombus vosnesenskii* (Hymenoptera: Apidae) pollinate greenhouse-grown tomatoes in western North America. J Econ Entomol. 2015:108:873–879. 10.1093/jee/tov078. 26470206

[evae207-B94] Sun C , Huang J, Wang Y, Zhao X, Su L, Thomas GWC, Zhao M, Zhang X, Jungreis I, Kellis M, et al Genus-wide characterization of bumblebee genomes provides insights into their evolution and variation in ecological and behavioral traits. Mol Biol Evol. 2020:38:486–501. 10.1093/molbev/msaa240. PMC7826183 32946576

[evae207-B95] Supek F , Bošnjak M, Škunca N, Šmuc T. Revigo summarizes and visualizes long lists of gene ontology terms. PLoS One. 2011:6:e21800. 10.1371/journal.pone.0021800. 21789182 PMC3138752

[evae207-B96] Theodorou P , Radzevičiūtė R, Kahnt B, Soro A, Grosse I, Paxton RJ. Genome-wide single nucleotide polymorphism scan suggests adaptation to urbanization in an important pollinator, the red-tailed bumblebee (*Bombus lapidarius L.*). Proc R Soc Lond B Biol Sci. 2018:285:1–9. 10.1098/rspb.2017.2806. PMC5936727 29669900

[evae207-B97] Walsh AT , Triant DA, Tourneau JJL, Elsik CG. Hymenoptera genome database: new genomes and annotation datasets for improved go enrichment and orthologue analyses. Nucleic Acids Res. 2021:50:D1032–D1039. 10.1093/nar/gkab1018. PMC8728238 34747465

[evae207-B98] Woodard SH . Bumble bee ecophysiology: integrating the changing environment and the organism. Curr Opin Insect Sci. 2017:22:101–108. 10.1016/j.cois.2017.06.001. 28805631

[evae207-B99] Yan H , Bonasio R, Simola DF, Liebig J, Berger SL, Reinberg D. DNA methylation in social insects: how epigenetics can control behavior and longevity. Annu Rev Entomol. 2015:60:435–452. 10.1146/annurev-ento-010814-020803. 25341091

[evae207-B100] Zhou W , Johnson BK, Morrison J, Beddows I, Eapen J, Katsman E, Semwal A, Habib WA, Heo L, Laird PW, et al BISCUIT: an efficient, standards-compliant tool suite for simultaneous genetic and epigenetic inference in bulk and single-cell studies. Nucleic Acids Res. 2024:52(6):e32. 10.1093/nar/gkae097. 38412294 PMC11014253

