# Supplementary material for: Examining the Effects of Environment, Geography, and Elevation on Patterns of DNA Methylation Across Populations of Two Widespread Bumble Bee Species
Source: Genome Biol Evol. 2024 Sep 27;16(10):evae207. doi: 10.1093/gbe/evae207 (PMC11474243; doi:10.1093/gbe/evae207)
Supplement: evae207_Supplementary_Data [file evae207_supplementary_data.docx]

**Supplemental Materials**

**Figures
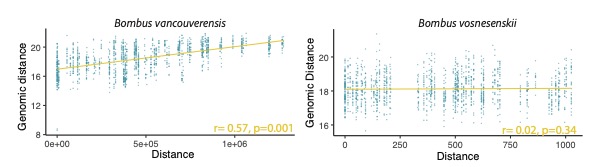
**

Figure S1: Relationship between genomic distance between individuals using the SNP dataset generated by biscuit versus the geographic distance between individuals.

**Tables**

| *B. vancouverensis* | | | | |
| --- | --- | --- | --- | --- |
| Site | Latitude | Longitude | Elevation | n |
| OR05.2021 | 42.07611 | -122.71781 | 2041.5504 | 4 |
| CA01.2021 | 41.35558 | -122.20754 | 2261.3112 | 4 |
| OR10.2021 | 44.224 | -121.87189 | 1466.088 | 2 |
| CA17.2015 | 37.21686 | -119.19571 | 7294 | 3 |
| CA20.2015 | 37.87672 | -119.35461 | 8587 | 6 |
| CA25.2015 | 39.23011 | -120.14201 | 7745 | 5 |
| OR03.2015 | 42.39619 | -122.20124 | 4901 | 4 |
| OR08.2015 | 45.33274 | -121.67026 | 5574 | 7 |
| CA33.2015 | 41.35381 | -122.23202 | 6953 | 4 |
| WA03.2016 | 48.58863 | -120.37342 | 2402 | 6 |
| WA06.2016 | 46.71925 | -120.82024 | 1770 | 2 |
| WA13.2016 | 48.6514 | -120.40205 | 5557 | 2 |
| OR06.2016 | 45.2559 | -121.71207 | 3435 | 4 |
| *B. vosnesenskii* | | | | |
| Site | Latitude | Longitude | Elevation | n |
| OR03.2021 | 45.6272 | -121.51741 | 218.8464 | 6 |
| OR06.2021 | 42.191856 | -122.66835 | 566.928 | 6 |
| OR11.2021 | 45.32446 | -121.65761 | 1573.6824 | 4 |
| CA06.2015 | 37.012 | -119.732 | 430 | 6 |
| CA10.2015 | 37.88836 | -120.52287 | 1044 | 4 |
| CA12.2015 | 40.50844 | -122.3218 | 454 | 5 |
| CA13.2015 | 36.61918 | -118.81165 | 7441 | 6 |
| CA15.2015 | 37.2023 | -119.21434 | 7372 | 4 |
| CA23.2015 | 38.32296 | -119.67812 | 8191 | 4 |
| CA03.2016 | 40.79306 | -122.88858 | 2569 | 5 |
| CA02.2014 | 41.36148 | -122.20068 | 7800 | 1 |
| OR10.2014 | 42.07605 | -122.71729 | 6742 | 4 |
| CA02.2015 | 36.8187 | -118.889 | 3840 | 3 |

Table S1: Summary of specimen information using in sampling. “n” refers to the number of samples collected in a given sampling locality.

| *B. vancouverensis* | | |
| --- | --- | --- |
|  | Genic v intergenic | exon v intron |
| total v. HVar | χ^2^= 35,571, df = 1, p-value < 2.2e-16 | χ^2^= 432,394, df = 1, p-value < 2.2e-16 |
| total v. Meth30 | χ^2^= 22,419, df = 1, p-value < 2.2e-16 | χ^2^= 231,299, df = 1, p-value < 2.2e-16 |
| HVar v. Meth30 | χ^2^= 2,701, df = 1, p-value < 2.2e-16 | χ^2^= 3,449, df = 1, p-value < 2.2e-16 |
| *B. vosnesenskii* | | |
|  | Genic v intergenic | exon v intron |
| total v. HVar | χ^2^= 100,341, df = 1, p-value < 2.2e-16 | χ^2^= 999,634, df = 1, p-value < 2.2e-16 |
| total v. Meth30 | χ^2^= 73,972, df = 1, p-value < 2.2e-16 | χ^2^= 600,642, df = 1, p-value < 2.2e-16 |
| HVar v. Meth30 | χ^2^= 12,156, df = 1, p-value < 2.2e-16 | χ^2^ 12,980, df = 1, p-value < 2.2e-16 |

Table S2: Results of Chi-squared tests using the SNP filtered datasets. Total refers to all CpG’s in the genome. HVar refers to CpG’s retained in variable dataset (CpG’s with greater than 2 standard deviations in percent methylation and recovered as methylated in at least four individuals). Meth30 refers to the CpG’s in the methylated dataset (CpGs with ≥ 30% methylation on average)

| *B. vancouverensis* | | | | | |
| --- | --- | --- | --- | --- | --- |
| Model | Inertia | R2 | p(>F) | Proportion of explainable variance | Proportion of total variance |
| Full model | 11,241,179 | 0.02094566 | 0.001 | 1 | 0.207101735 |
| Climate | 6,968,334 | 0.01296278 | 0.001 | 0.619893518 | 0.128381023 |
| Geography | 2,894,320 | 0.00736914 | 0.002 | 0.257474772 | 0.053323472 |
| Structure | 1,259,843 | 0.00046279 | 0.434 | 0.11207392 | 0.023210703 |
| Confounded | 118,682 |  |  | 0.010557789 | 0.002186536 |
| Total unexpected | 43,037,357 |  |  |  | 0.792898265 |
| Total inertia | 54,278,536 |  |  |  | 1 |
|  |  |  |  |  |  |
| *B. vosnesenskii* | | | | | |
| Model | Inertia | R2 | p(>F) | Proportion of explainable variance | Proportion of toral variance |
| Full model | 15,118,877 | 0.01829305 | 0.001 | 1 | 0.17364343 |
| Climate | 8,757,893 | 0.01230993 | 0.001 | 0.579268751 | 0.100586213 |
| Geography | 4,278,363 | 0.00532458 | 0.008 | 0.282981534 | 0.049137884 |
| Structure | 1,869,376 | -0.0002584 | 0.606 | 0.123645162 | 0.02147017 |
| Confounded | 213,245 |  |  | 0.014104553 | 0.002449163 |
| Total unexpected | 71,949,646 |  |  |  | 0.82635657 |
| Total inertia | 87,068,523 |  |  |  | 1 |

Table S3: Summarized results of partial redundancy analyses (pRDA) data for both focal species using the SNP filtered dataset. Inertia is synonymous with variance. Model significance is reported in the p(>F) column. Proportion of explainable variance is the ratio between the inertia of a given model and the full model. The proportion of total variance is the ratio between inertia accounted for in a given model and the total inertia in the dataset.
